# Delving into Molecular Pathways: Analyzing the Mechanisms of Action of Monoclonal Antibodies Integrated in IMGT/mAb-DB for Myasthenia Gravis

**DOI:** 10.3390/vaccines11121756

**Published:** 2023-11-26

**Authors:** Rebecca Golfinopoulou, Véronique Giudicelli, Taciana Manso, Sofia Kossida

**Affiliations:** 1 IMGT, The International ImMunoGeneTics Information System, National Center for Scientific Research (CNRS), Institute of Human Genetics (IGH), University of Montpellier (UM), 34090 Montpellier, France; r.golfinopoulou@aua.gr (R.G.); veronique.giudicelli@igh.cnrs.fr (V.G.); 2 Department of Biotechnology, School of Applied Biology and Biotechnology, Agricultural University of Athens, 11855 Athens, Greece

**Keywords:** monoclonal antibodies, myasthenia gravis, bioinformatics, mechanism of action, IMGT

## Abstract

Background: Myasthenia Gravis (MG) is a rare autoimmune disease presenting with auto-antibodies that affect the neuromuscular junction. In addition to symptomatic treatment options, novel therapeutics include monoclonal antibodies (mAbs). IMGT^®^, the international ImMunoGeneTics information system^®^, extends the characterization of therapeutic antibodies with a systematic description of their mechanisms of action (MOA) and makes them available through its database for mAbs and fusion proteins, IMGT/mAb-DB. Methods: Using available literature data combined with amino acid sequence analyses from mAbs managed in IMGT/2Dstructure-DB, the IMGT^®^ protein database, biocuration allowed us to define in a standardized way descriptions of MOAs of mAbs that target molecules towards MG treatment. Results: New therapeutic targets include FcRn and molecules such as CD38, CD40, CD19, MS4A1, and interleukin-6 receptor. A standardized graphical representation of the MOAs of selected mAbs was created and integrated within IMGT/mAb-DB. The main mechanisms involved in these mAbs are either blocking or neutralizing. Therapies directed to B cell depletion and plasma cells have a blocking MOA with an immunosuppressant effect along with Fc-effector function (MS4A1, CD38) or FcγRIIb engager effect (CD19). Monoclonal antibodies targeting the complement also have a blocking MOA with a complement inhibitor effect, and treatments targeting T cells have a blocking MOA with an immunosuppressant effect (CD40) and Fc-effector function (IL6R). On the other hand, FcRn antagonists present a neutralizing MOA with an FcRn inhibitor effect. Conclusion: The MOA of each new mAb needs to be considered in association with the immunopathogenesis of each of the subtypes of MG in order to integrate the new mAbs as a viable and safe option in the therapy decision process. In IMGT/mAb-DB, mAbs for MG are characterized by their sequence, domains, and chains, and their MOA is described.

## 1. Introduction

**Figure 1:**
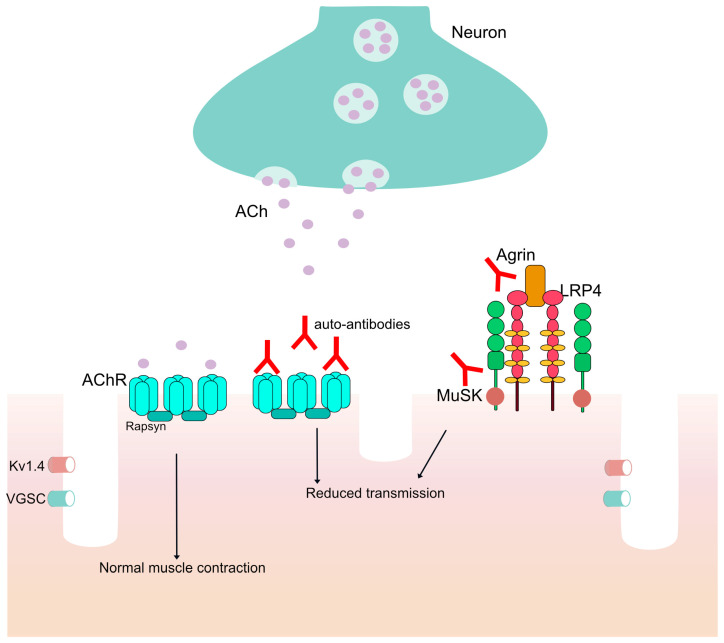
The neuromuscular junction (NMJ). ACh is released, and normal muscle contraction occurs. In patients with auto-antibodies against proteins in the NMJ, there is reduced transmission of the signal from the neuron to the muscles through the voltage-gated potassium channel (Kv1.4) and the voltage-gated sodium channel (VGSC).

Myasthenia Gravis (MG) is a rare chronic autoimmune disease with fluctuating symptoms deriving from the presence of auto-antibodies, immunoglobulins directed against an individual’s own proteins at the post-synaptic neuromuscular junction (NMJ) of skeletal muscles (Figure 1) [1]. MG can be characterized as ocular or generalized with a spectrum of symptoms manifesting in patients, including extraocular symptoms (diplopia and/or ptosis) in both ocular and generalized MG (gMG) and muscle weakness, dysphagia and, in some cases, respiratory weakness in gMG, and their clinical severance may vary from mild to potentially life-threatening. The disease can have either an early onset (EOMG) or late-onset MG (LOMG) and can be further subcategorized by the type of antibodies the patient presents, including auto-antibodies against the acetylcholine receptor (AChR), muscle-specific kinase (MuSK), lipoprotein related protein 4 (LRP4), agrin and rapsyn [2,3,4,5,6].

AChR antibodies are very specific to Myasthenia Gravis and are present in about 80–85% of patients [7]. Their presence is verified through serological testing and, combined with the basic symptom of muscle weakness, verifies the disease. The AChR antibodies found in Myasthenia Gravis patients may be of two subclasses, IgG1 and IgG3. These isotypes can bind to the post-synaptic AChR and lead to a complement cascade, formation of the membrane attack complex (MAC), and limited or reduced signal transmission to the muscles [8]. In patients with anti-MuSK antibodies, the pathogenesis is different, as the antibodies belong to the IgG4 subclass with limited C1q binding. These antibodies prevent AChR clustering by masking the site of regular MuSK-LRP4 interaction [9]. A small percentage of patients are seronegative but have a similar clinical presentation, and antibodies against LRP4 have been reported to be detected in 1–5% of MG patients [10]. These antibodies can co-exist with anti-AChR or anti-MuSK antibodies and can be detected in other autoimmune diseases, such as neuromyelitis optica (NMO) and multiple sclerosis (MS) [11,12].

An important role in the disease is played by the thymus gland. Thymus hyperplasia is present in more than 70% of patients, and about 10–15% of them have a thymoma caused by abnormal development of the epithelial cells [13]. More than 50% of anti-AChR positive patients have a hyperplastic thymus with the production of B cells, AChR antibodies, and the presence of anti-AChR reactive T cells. Although regulatory T (Treg) cells CD4+ and CD25+ have a significant role in controlling the immune and autoimmune response, the exact mechanism by which the thymus is involved in the attack against AChRs is not yet clear [14].

Currently, there are only symptomatic treatment options, including pyridostigmine and other acetylcholinesterases and corticosteroids, as the primary options for patients. Pyridostigmine bromide is an acetylcholinesterase inhibitor (AChEI) and is the first-line agent for symptomatic treatment of the disease, followed by neostigmine [15]. Corticosteroids inhibit T cells and monocyte–macrophage activation and are part of the primary course of treatment for most patients [16]. Other immunosuppressants include Azathioprine, Mycophenolate mofetil (MMF), Cyclosporine, Cyclophosphamide, Methotrexate, and Tacrolimus [15]. In cases of myasthenic crisis, intravenous immunoglobulins (IVIG) and therapeutic plasma exchange (PLEX) offer a rapid response to the patient’s organism. MG treatment that includes immunosuppressants, such as azathioprine, entails long-term immunosuppression [17,18].

Monoclonal antibodies (mAbs) and fusion proteins have been employed as immunotherapy against a specific autoimmune component in the disease by different mechanisms of action (MOA). To date, there are more than 300 mAbs and fusion proteins assigned by the World Health Organization’s (WHO) International Nonproprietary Names (INN) Program in the field of autoimmune diseases, of which 66 have been approved by the U.S. Food and Drug Administration (FDA) and/or European Medicines Agency (EMA). Eculizumab was the first mAb approved to treat MG, with the initial study involving 14 patients indicating a significant improvement in the patient’s daily activities and a 75% reduction in the frequency of exacerbation [19]. Eculizumab’s effectiveness in preventing the formation of MAC was previously evaluated in patients with atypical hemolytic uremic syndrome (aHUS) [20] and paroxysmal nocturnal hemoglobinuria (PNH) [21] and was also effective in reducing relapse risk in neuromyelitis optica spectrum disorder (NMOSD) [22]. At present, there are four more FDA-approved mAbs used for the treatment of MG (efgartigimod alfa, ravulizumab, rozanolixizumab, and rituximab) that are analyzed in the Results section.

Currently, IMGT^®^, the international ImMunoGeneTics information system^®^ (http://www.imgt.org) [23], extends the characterization of therapeutic antibodies with the description of their mechanisms of action and makes them available through its database for mAbs and fusion proteins, IMGT/mAb-DB [24]. In this study, we analyze fifteen mAbs and fusion proteins developed to treat Myasthenia Gravis and autoimmune diseases with auto-antibodies. The aim is to describe how monoclonal antibodies can be effective in MG, highlighting how engineered antibodies could potentially prevent pathogenic auto-antibodies from binding to the receptors and minimize symptoms.

## 2. Methods

Literature searches were carried out to establish the mAbs targeting specific molecules towards MG treatment. The terms “monoclonal antibodies in myasthenia gravis” and “myasthenia gravis treatment” were searched in the MEDLINE and PubMed databases. The mAbs developed to treat MG have been retrieved in the “Clinical indication” field in the IMGT/mAb-DB query page and were categorized by their target (C5, FCGRT, CD40, CD19, MS4A1, IL6-IL6R, and CD38). The mAbs retrieved were studied extensively using a literature search, and the MOA of each mAb (referred to by its INN) is described in this study. The literature search included clinical trials, pharmacokinetics/pharmacodynamics, molecular structure, and literature regarding each target identified and each mAb in other diseases. To synthesize each mAb MOA, the scientific literature relevant to the target and mAb was examined. For previously studied targets and antibodies with a well-defined MOA, data were extracted from the literature and used to standardize the description of the MOA. For the unstudied antibodies, data from the literature were used to infer the description of the mAbs function in MG and other autoimmune diseases, and a suggested MOA was derived, while the phrase “proposed by IMGT” was added in the MOA description.

Each MOA description is accompanied by a standardized schematic depiction created with the AFFINITY Designer tool (Serif, RRID: SCR_016952, version 1.10.5.1342). Keywords to best describe the mechanism and the anticipated immune response are included along with the HGNC gene name for the target and its abbreviation. The AA sequences of each mAb were carefully examined using the IMGT/2Dstructure-DB and the IMGT/DomainGapAlign tool [25] for mutation identifications in the Fc region, and their effects were included in the standardized description, according to IMGT-engineered variants [26]. 

## 3. Results

### 3.1. Monoclonal Antibodies for Complement Inhibition

During the 1970s, Engel and his colleagues were the first to identify the role of the complement in the pathophysiology of MG by visualizing antibodies against AChR, C3, and MAC in the post-junctional membrane in patients with MG [27]. Subsequent important findings revealed a decrease in C3 and C4 levels along with an increase in terminal complement components (C5b-C9) in the sera of MG patients [28,29]. The significant role of the complement system in both innate and antibody-mediated immunity in MG has been primarily demonstrated through experimental autoimmune Myasthenia Gravis (EAMG) animal models. These models have shown that the administration of anti-complement antibodies and inhibition of MAC formation can protect against the development of muscle weakness. Additionally, complement inhibition in EAMG models has the potential to prevent disease induction and reverse its progression [30]. The proinflammatory and prothrombotic effects of C5a and C5b, including chemotaxis of inflammatory cells and MAC-mediated cell activation and lysis, are prevented by blocking terminal complement activation at the complement 5 (C5) point. The formation of MAC and/or the release of C5a from unregulated complement activity causes localized damage of the post-synaptic membrane of the NMJ, which makes terminal complement inhibition a potentially useful treatment approach for illnesses such as MG [31]. Some mAbs for complement inhibition have been developed to treat different autoimmune diseases comprising MG. IMGT/mAb-DB includes nine mAbs assigned by INN, two of which have been approved by the FDA and/or EMA, namely eculizumab (SOLIRIS™, IMGT/mAb-DB ID: mAbID 37) and ravulizumab (ULTOMIRIS™, mAbID 674) (Table 1).

**Figure 2:**
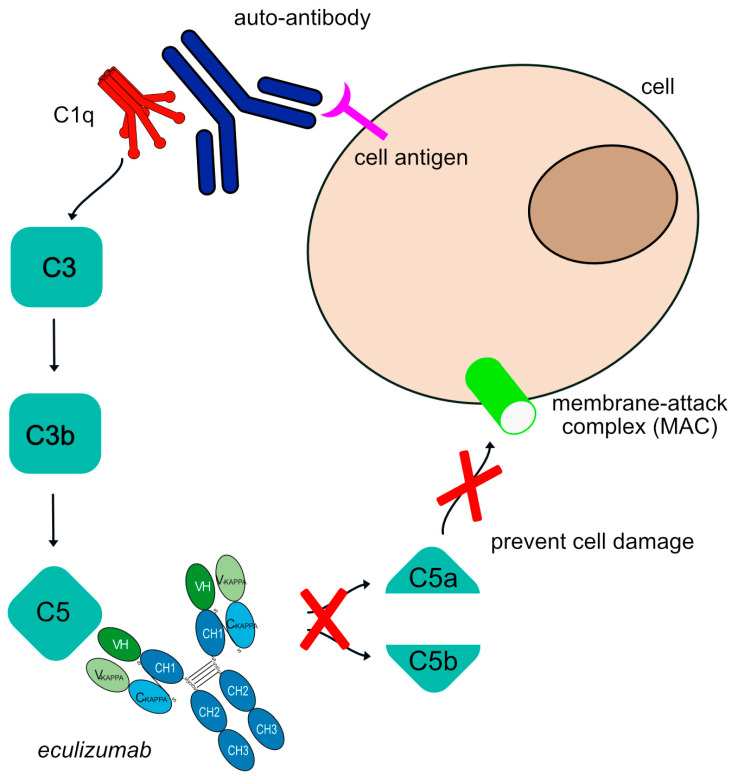
Eculizumab mechanism of action. The human auto-antibodies bind antigens and allow C1q binding and classical complement cascade activation. This is followed by some cleavages to produce a C3 molecule that is cleaved to form C3a and C3b and cleave C5 into C5a and C5b. This allows further recruitment and activation of other complement components to form the membrane attack complex (MAC). The complement cascade is blocked by eculizumab as it targets the C5 molecule, inhibiting the production of molecules C5a and MAC. Eculizumab is an IgG2–IgG4 hybrid antibody to reduce complement-dependent cytotoxicity (CDC) and FcγR effector properties. Mechanism of action: Blocking. Effect: Complement inhibitor. (mAbID 37).

Eculizumab is a humanized IgG2–G4-kappa antibody that targets complement 5 (C5), previously established as therapy for disorders such as hemolytic uremic syndrome and paroxysmal nocturnal hemoglobinuria [32]. In 2017, eculizumab was approved to treat gMG in adult patients who are anti-acetylcholine receptor (AchR) antibody positive. Eculizumab targets and binds to C5, and once bound to its target, the mAb stops the progression of the complement cascade regardless of the stimuli. Additionally, stopping the cleavage of C5 efficiently blocks the production of potent proinflammatory molecule C5a and the cell lytic terminal complement complex (TCC). It is significant that the critical immunoprotective and immunoregulatory functions of the proximal components of complement are preserved by C5 blockade, as these upstream proteins of C5 are essential for microbial opsonization (they lead to C3b-mediated opsonization) and immune complex clearance [33]. The Fc region of eculizumab is a hybrid of IgG2 and IgG4 isotypes to reduce both complement activation and FcγR binding. By specifically targeting the C5 complement protein, the formation of C5b-9 or the membrane attack complex (MAC) is intercepted, preventing cell damage. Therefore, the antibody’s MOA is a “Blocking-Complement inhibitor” (Figure 2).

Ravulizumab is a humanized IgG2–G4 hybrid antibody that also targets complement protein C5 with a prolonged half-life due to mutations in the Fc region to enhance FcRn binding (G4v24 CH3 L107, S114) [34]. Currently, a phase 3 randomized, double-blind, Placebo-Controlled Study to evaluate the safety and efficacy of ravulizumab in adult patients with gMG is ongoing (clinicaltrials.gov NCT03920293) [35].

A clinical trial to compare eculizumab versus ravulizumab in adult patients with PNH showed that treatment every 8 weeks with ravulizumab was non-inferior compared with every 2 weeks treatment with eculizumab for the primary endpoint (percentage change in LDH in PNH patients) and for additional secondary endpoints (transfusion avoidance (TA), breakthrough hemolysis (BTH), stabilized hemoglobin (HGB-S), and FACIT-Fatigue score) [36]. 

Different mAb constructs have been developed to increase the treatment efficacy. Gefurulimab (mAbID 1253), a bispecific VH-VH mAb that binds to C5 (to inhibit complement cascade) and albumin (to extend antibody half-life), are currently under clinical development for the treatment of gMG, along with dermatomyositis and proteinuria. A phase 3 clinical trial to evaluate the safety and efficacy of the mAb in patients with gMG is currently recruiting patients (ClinicalTrials.gov NCT05556096) and is estimated to be completed in 2027. Regarding MG, pozelimab, an IgG4 antibody anti-C5, is currently recruiting for phase 3 to examine the efficacy and safety in MG (ClinicalTrials.gov NCT05070858). This study examines a combination therapy of the mAb with Cemdisiran, an N-acetylgalactosamine (GalNAc) conjugated RNA interference (RNAi) therapeutic. This C5 inhibitor has been evaluated for paroxysmal nocturnal hemoglobinuria (PNH), CD55-deficient protein-losing enteropathy, and CHAPLE disease [37,38]. Other C5 inhibitors designed and studied for other diseases may, in the future, be studied for possible therapeutic effects in MG patients.

### 3.2. Monoclonal Antibodies as FcRn Antagonists

Fc gamma (γ) receptors (FcγR) are members of a large family of proteins that now includes cytoplasmic glycoproteins, atypical intracellular receptors, and traditional membrane-bound surface receptors. The fragment crystallizable neonatal receptor (FcRn, FCGRT), one of the atypical FcγRs, binds albumin and IgG, and it protects IgG from degradation by releasing it back into the serum and expanding the IgG life cycle [39]. MG, as an antibody-mediated disease, can be treated by eliminating the number of pathogenic IgG in the patient’s serum. Therefore, blocking this physiologic FcRn mechanism can be proven beneficial through the administration of mAbs that target FcRn, leading to increased antibody catabolism and reducing the levels of pathological auto-antibodies [40].

**Figure 3:**
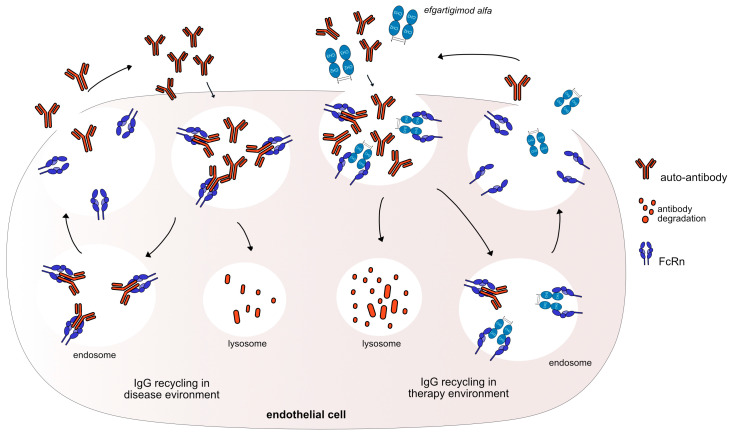
Efgartigimod alfa mechanism of action. Auto-antibodies bind to FcRn to recycle them and keep them in the body. Efgartigimod alfa binds with high affinity to FcRn (G1v46 CH3 K113, F114), keeping FcRn occupied and neutralizing the antibody recycling. Thus, efgartigimod alfa leads to increased IgG catabolism, resulting in a reduction in the available IgG. The auto-antibodies could be destroyed and removed from the body. Mechanism: Neutralizing. Effect: FcRn inhibitor. (mAbID 731).

IMGT/mAb-DB includes one fusion protein, efgartigimod alfa (VYVGART™, mAbID 731), that was approved in 2021 by the FDA to treat gMG patients, and four mAbs (Table 2). Efgartigimod alfa is a humanized IgG1 Fc-fragment that targets and binds to the FcRn and outcompetes endogenous IgG binding, leading to increased IgG catabolism, resulting in a reduction in the available IgGs. In addition, efgartigimod alfa has three mutations in the CH2 domain and two amino-acid changes in the CH3 domain (G1v96 CH2 Y15.1, T16, E18; CH3 K113, F114) to increase FCGRT (FcRn) binding (without pH dependency) and its half-life (Figure 3).

In addition to efgartigimod alfa, there are four mAbs targeting FcRn included in IMGT/mAb-DB (Table 2). Nipocalimab (mAbID 1020), a human aglycosylated mAb via one mutation in the CH2 domain (G1v29 A84.4) which prevents binding to FcyRs and reduces side effects, binds to FcRn with a picomolar affinity at both endosomal pH 6.0 and extracellular pH 7.6, permitting FcRn occupancy throughout the recycling pathway [41]. Rozanolixizumab (Rystiggo^®^, mAbID 642) has been recently approved by the FDA for subcutaneous infusion following the results of the MycarinG Phase 3 study (NCT03971422) and is the only FDA-approved treatment in adults for both anti-AChR and anti-MuSK antibody-positive gMG [42].

### 3.3. Monoclonal Antibodies for B Cell Depletion

Immunosuppressants used in MG usually have a delayed positive effect on the disease, and patients with refractory MG have been proven through studies to benefit from treatments such as rituximab that may help in inducing a stable remission [43,44,45,46,47]. B cells play a key role in host defense by secreting antibodies, which are the components of the humoral adaptive immune system [48]. B-cell-specific surface antigens MS4A1 and CD19 are used as targets in many autoimmune diseases, including MG, with MS4A1 surface molecule (aka CD20) being the most reliable marker of B lymphocytes and CD19 is expressed on a broader group of cells [49]. MS4A1 is thought to function for B cell activation, proliferation, and differentiation and is associated with a variety of B cell surface molecules [50,51,52]. It is expressed during B cell differentiation from pre-B cells to the stage of plasmablasts, while CD19 molecules start at the late pro-B cell stage (prior to the expression of MS4A1) and are maintained throughout B cell activation until differentiation into plasma cells when CD19 expression is partially or completely lost [53]. 

**Figure 4:**
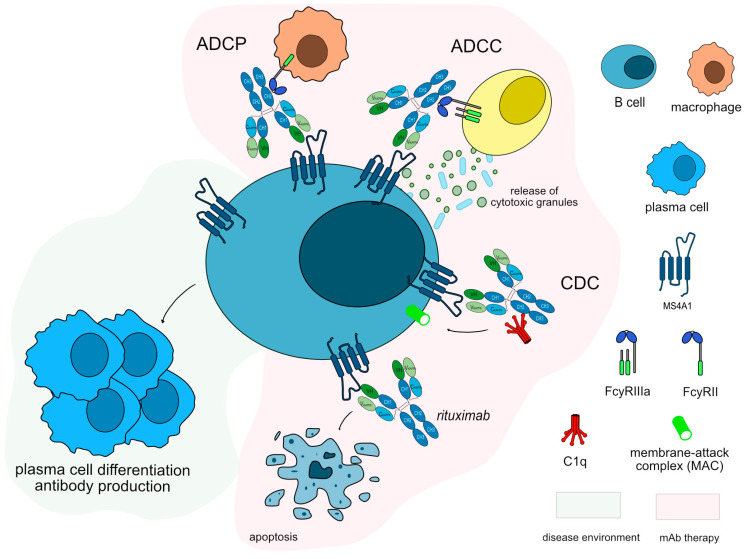
Rituximab mechanism of action. Rituximab targets and binds to MS4A1 expressed on the surface of B cells. Once bound to its target, rituximab induces apoptosis of CD20+ cells, resulting in the depletion of B-cells. Rituximab is an IgG1-kappa antibody able to mediate complement-dependent cytotoxicity (CDC), antibody-dependent cellular cytotoxicity (ADCC), and antibody-dependent cellular phagocytosis (ADCP) against CD20+ B cells to completely deplete this population. Mechanism of action: Blocking. Effect: Immunosuppressant, Fc-effector function. (mAbID 161).

IMGT/mAb-DB includes five mAbs targeting MS4A1 and two mAbs targeting CD19 for autoimmune diseases, of which four and one, respectively, have been approved by the FDA and/or EMA. Rituximab (MABTHERA^®^, RITUXAN^®^, mAbID 161) is a chimeric murine/human antibody binding to MS4A1 that depletes B cells by apoptosis and by indirect effects such as antibody-dependent cellular cytotoxicity (ADCC), CDC and antibody-dependent cellular phagocytosis (ADCP) (Figure 4). Rituximab is one of the first mAb therapies explored for MG due to the role of B cells in the disease pathogenesis. Its MOA is blocking with an immunosuppressant effect and Fc-effector function to deplete B cells efficiently. It has been shown to be an effective treatment also for anti-MuSK MG patients [54]. Rituximab is mentioned as an option when conventional MG medicine fails, or there is a patient intolerance towards them, according to the 2021 international consensus guidelines on MG [55]. Rituximab is also being used in other autoimmune diseases, including rheumatoid arthritis (RA) [56], multiple sclerosis (MS) [57], and neuromyelitis optica (NMO) [58], showing the significance of targeting MS4A1 and B cells.

Among the other four monoclonal antibodies (mAbs) directed against MS4A1 mentioned in IMGT/mAb-DB, there exists only a single instance where a patient suffering from refractory AChR-MG exhibited a positive response to ofatumumab. The resemblance of ofatumumab’s mechanism of action to that of rituximab, along with its heightened binding affinity for a distinct subtype of receptors, implies its potential effectiveness in managing MG as well [59,60] (Table 3). Nonetheless, it’s worth emphasizing that none of these antibodies have been incorporated into clinical trials with a specific focus on MG. The implementation of such trials has the potential to yield valuable insights into the potential utility of these antibodies for MG treatment [59,61].

**Figure 5:**
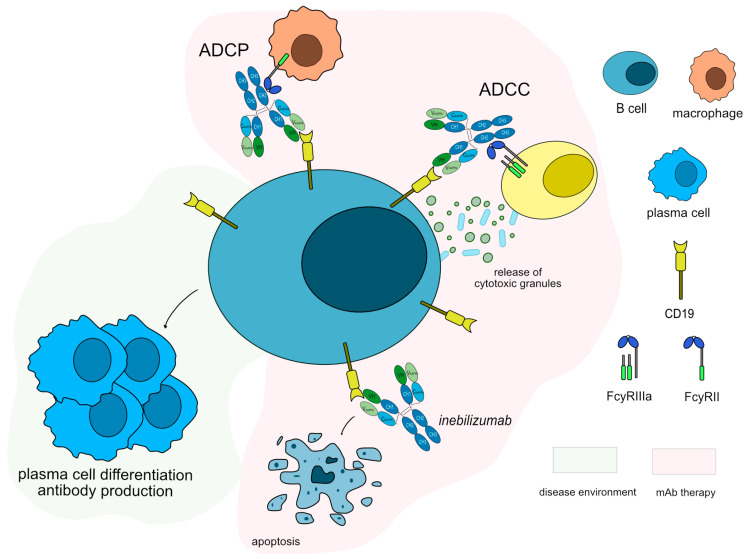
Inebilizumab mechanism of action. Inebilizumab targets and binds to CD19 antigen expressed in B cells. Once bound to its target, inebilizumab induces apoptosis of CD19+ cells, resulting in the depletion of B-cells. Inebilizumab is an IgG1-kappa antibody able to mediate antibody-dependent cellular cytotoxicity (ADCC) and antibody-dependent cellular phagocytosis (ADCP) against CD19+ B cells to completely deplete this population, suppress inflammatory responses and impair B-cell-dependent T-cell activation. Mechanism of action: Blocking. Effect: Immunosuppressant, Fc-effector function. (mAbID 553).

Anti-CD19 monoclonal antibodies have been tested in both anti-AChR and anti-MuSK antibody-positive patients. Inebilizumab (UPLIZNA^®^, mAbID 553), a humanized antibody that binds and depletes B-cells that express CD19 molecule [62], is in phase III clinical trial with approximately 270 participants and an estimated completion date by the end of 2024 (NCT04524273) [63]. Inebilizumab targets and binds to CD19 antigen, which is continuously and stably expressed on all stages of B lineage differentiation on the surface of B cell lymphocytes [53]. Once bound to CD19, inebilizumab depletes circulating CD19+ B cells, thus suppressing inflammatory responses and impairing B-cell-dependent T-cell activation. Inebilizumab is an IgG1-kappa afucosylated mAb with an increased ability to bind to FcγRIIIa, and it induces a strong ADCC for enhanced efficacy against plasma cells and auto-antibodies production (Figure 5). The MOA of inebilizumab is “Blocking–Immunosuppressant, Fc-effector function.”

Anti-CD19 agents may have a promising future as the transmembrane protein CD19 is expressed on a vast majority of B cells, making it a potentially good target for mAb-mediated immunotherapy. A comparison between inebilizumab and rituximab showed three key differences [64,65]. First, rituximab-mediated B-cell depletion involved both macrophages and complement, whereas inebilizumab-mediated B-cell depletion involved macrophages but not complement. Secondly, compared with rituximab, inebilizumab significantly reduced the number of B cells in the bone marrow. Third, the duration of cell depletion was greater following inebilizumab treatment compared with after rituximab treatment. Combining inebilizumab with rituximab treatment further sustained B-cell depletion, demonstrating the possibility of synergistic effects between the two B-cell-depleting treatments [64].

### 3.4. Therapies Directed to Plasma Cell

A promising new option for the treatment of the disease based on experimental studies is therapeutic agents that aim for a reduction in plasma cells that produce pathogenic auto-antibodies in patients with MG. In this way, the surface markers of the long-lived memory plasma cells may be an attractive therapeutic approach. CD38, a type II transmembrane glycoprotein that synthesizes and hydrolyzes cyclic adenosine 5’-diphosphate-ribose, is expressed in Ig-secreting thymocytes and plasma cells and has multiple functions, including receptor-mediated adhesion, signaling, and modulation of cyclase and hydrolase activity [66].

**Figure 6:**
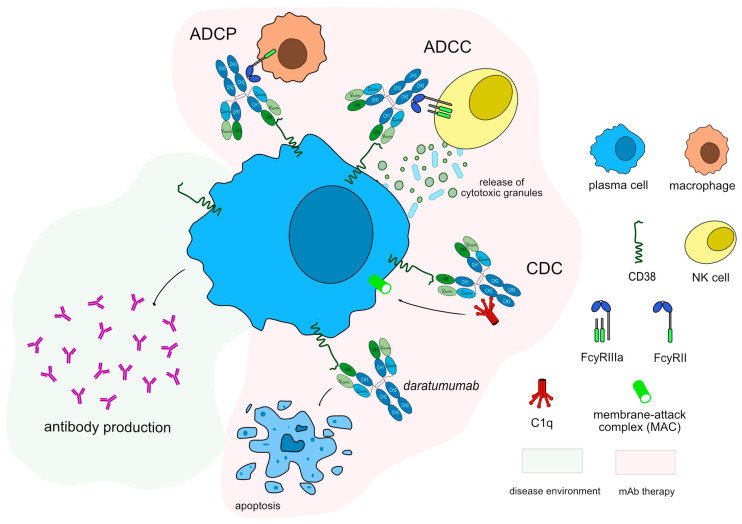
Daratumumab mechanism of action. Daratumumab targets and binds to CD38 expressed in plasma cells. Once bound to its target, daratumumab induces apoptosis of CD38+ cells, resulting in the depletion of plasma cells. Daratumumab is an IgG1-kappa antibody able to mediate complement-dependent cytotoxicity (CDC), antibody-dependent cellular cytotoxicity (ADCC), and antibody-dependent cellular phagocytosis (ADCP) against CD38+ cells to completely deplete this population. Mechanism of action: Blocking. Effect: Immunosuppressant, Fc-effector function. (mAbID 301).

IMGT/mAb-DB contains six mAbs against CD38, two of which have been approved by the FDA for cancer treatment; however, none reached the clinic yet in the field of autoimmune diseases (Table 4). Daratumumab (DARZALEX™/DARZALEX FASPRO™, mAbID 301) is a humanized IgG1-kappa antibody that targets CD38 inducing apoptosis by dysregulating the CD38 enzymatic function and indirectly through Fc mediated cross-linking as well as by immune-mediated cell lysis through CDC, ADCC, and ADCP. Thus, its MOA is “Blocking–Fc-effector function–Immunosuppressant’’ to deplete CD38-expressing cells (Figure 6). This drug has been approved as a therapeutic option for multiple myeloma and could be an alternative option in order to reduce the number of plasma cells in MG patients. A recent study for MG suggests that daratumumab may be a promising treatment for refractory auto-antibody-mediated neurological disorders [67]. Similarly, mezagitamab (mAbID 882) is in a phase II clinical trial that was recently completed (clinicaltrials.gov NCT04159805), aimed to study if patients with generalized MG have side effects when administered low or high doses of mezagitamab.

Plasma cells are characterized by highly active immunoglobulin synthesis and, in these cells, accumulation of misfolded proteins and apoptosis resulting from the inhibition of the proteasome function [60]. In B-cell neoplasms, this strategy has been proven to be effective as a treatment, and the proteasome inhibitor Bortezomib has been studied for MG as it depletes both short- and long-lived plasma cells [68,69].

**Table 4:** mAbs studied in this work based on targets related to MG and other autoimmune diseases.

INN mAbs	Target	Receptor Identification	IMGT MOA **	Clinical Indication	IMGT Variants	FDA/EMA Approval
eculizumab * (mAbID 37)	C5	IgG2–G4-kappa	Blocking ** Complement inhibitor	Dermatomyositis, Nephritis, Paroxysmal nocturnal hemoglobinuria (PNH), Psoriasis, Rheumatoid Arthritis (RA), Atypical hemolytic uremic syndrome (aHUS), Neuromyelitis optica (NMO), Myasthenia Gravis (MG), Kidney transplantation treatment of antibody-mediated rejection (AMR), Kidney transplantation prevention of delayed graft function (DGF)		EMA: October 2003 FDA: March 2007
ravulizumab * (mAbID 674)		IgG2–G4-kappa		PNH|Atypical hemolytic uremic syndrome|MG	**G4v24** CH3 L107, S114 Half-life extension	EMA: May 216 FDA: December 2018
crovalimab (mAbID 783)		IgG1-kappa		PNH	**G1v94** CH2 R1.2, R1.1, K3, G110, S115, S116 ADCC and CDC reduction **G1v100** CH3 R118, E120 decrease Rheumatoid factor (RF) binding Fc variants with enhanced FcRn binding **G1v85** CH3 L107, A114 half life extensions	
gefurulimab *, *** (mAbID 1253)		VH–VH’		Complement component deficiency|MG		
tesidolumab (mAbID 535)		IgG1-lambda2		Age-related macular degeneration (AMD)|Choroiditis	**G1v14** CH2 A1.3, A1.2 ADCC and CDC reduction	
vilobelimab *** (mAbID 1038)		IgG4-kappa		Hidradenitis suppurativa | Inflammation		
olendalizumab (mAbID 585)		IgG2–G4-kappa		Graft-versus-host disease (GvHD)|Antiphospholipid syndrome (APS)		
pozelimab * (mAbID 898)		IgG4-kappa		PNH|MG	**G4v5** h P10 Half-IG exchange reduction	
efgartigimod alfa * (mAbID 731)	FCGRT	Fc-gamma1	Neutralizing ** FcRn inhibitor	MG|Primary immune thrombocytopenia (ITP)|Chronic inflammatory demyelinating polyneuropathy (CIDP)	**G1v96** CH2 Y15.1, T16, E18; CH3 K113, F114 Half-life extension without pH dependency	FDA: December 2021
batoclimab * (mAbID 943)		IgG1-lambda2		Autoimmune diseases|MG	**G1v14** CH2 A1.3, A1.2 ADCC and CDC reduction	
rozanolixizumab * (mAbID 943)		IgG4-kappa		MG|Thrombocytopenia/Immune thrombocytopenia (ITP)	**G4v5** h P10 Half-IG exchange reduction	FDA: April 2018
nipocalimab * (mAbID 1020)		IgG1-lambda3		Autoimmune diseases|MG	**G1v29** CH2 A84.4 No N-glycosylation site ADCC reduction	
orilanolimab (mAbID 854)		IgG4-kappa		Autoimmune diseases| Pemphigus vulgaris (PV)| Warm antibody autoimmune hemolytic anemia	**G4v5** h P10 Half-IG exchange reduction	
iscalimab *,*** (mAbID 799)	CD40	IgG1-kappa	Blocking ** Immunosuppressant	Psoriasis|Kindey transplant rejection|MG|Sjögren’s syndrome (SjS)|Graves’ orbitopathy (GO)	**G1v29** CH2 A84.4 No N-glycosylation site ADCC reduction	
bleselumab (mAbID 563)		IgG4-kappa		Psoriasis|Organ transplant immunological rejection suppression	**G4v5** h P10 Half-IG exchange reduction **G4v3** CH2 E1.2 ADCC and CDC reduction	
ravagalimab (mAbID 806)		IgG1-kappa		Crohn’s disease (CD)	**G1v14** CH2 A1.3, A1.2 ADCC and CDC reduction **G1v42** CH2 Q14; CH3 L107 Half-life extension	
inebilizumab *,*** (mAbID 553)	CD19	IgG1-kappa	Blocking ** Immunosuppressant, Fc-effector function	MG|Multiple sclerosis (MS)|NMO|MG Scleroderma| Neuromyelitis optica spectrum disorder| Chronic lymphocytic leukemia (CLL)| Lymphoma diffuse large B cell (DLBCL)		FDA: February 2016
obexelimab (mAbID 518)		IgG1-kappa		Autoimmune diseases| RA|Systemic lupus erythematosus (SLE)| IgG4-related disease (IgG4-RD)	**G1v25** CH2 E29, F113 B cell inhibition	
rituximab * (mAbID 161)	MS4A1 (CD20)	IgG1-kappa	Blocking ** Immunosuppressant, Fc-effector function	CLL (CD20-positive, in combination with fludaraline and cyclophosphamide (FC))| Solid organ transplantation| RA|MG DLBCL (in combination with hyaluronidase)| Wegener’s Granulomatosis (WG) and Microscopic Polygamiitis (MPA), in combination with glucocorticoids| Non-Hodgkin’s lymphoma (NHL), follicular CD20 positive, relapsed or refractory low grade|PV| Chronic focal encephalitis (CFE)|Waldenstrom macroglobulinemia (WM)		E EMA: June 1998 FDA: November 1997
ofatumumab * (mAbID 194)		IgG1-kappa		CLL|NHL|RA|MS (relapsing remitting)| Lymphoma follicular (LF)|NMO|PV|MG		EMA: April 2010 (withdrawn) FDA: October 2009
ublituximab (mAbID 372)		IgG1-kappa		CLL|DLBCL|NMO|MS, relapsing-remitting		FDA: August 2016
ocrelizumab (mAbID 227)		IgG1-kappa		RA|SLE|MS| Lupus Nephritis| Primary progressive multiple sclerosis (PPMS)		FDA: March 2017
divozilimab (mAbID 1060)		IgG1-kappa		MS, relapsing-remitting|Systemic Scleroderma|NMOSD		
tocilizumab * (mAbID 96)	IL6R	IgG1-kappa	Blocking ** Immunosuppressant	Lymphoproliferative disorder giant lymph node hyperplasia (Castleman’s disease)|Multiple myeloma (MM)|RA|Systemic juvenile idiopathic arthritis (SJIA)|Systemic sclerosis| Cytokine Release Syndrome (CRS)|NMO|Large-vessel vasculitis Giant cell arteritis| Polyarticular Juvenile Idiopathic Arthritis (PJIA)|Severe acute respiratory syndrome coronavirus 2 (SARS-CoV-2) infection (COVID-19)|MG		EMA: January 2009 FDA: January 2010
satralizumab (mAbID 586)		IgG2-kappa		NMO		EMA: June 2016 FDA: August 2020
levilimab *** (mAbID 887)		IgG1-kappa		RA	**G1v76** CH2 P1.4, V1.3, A1.2 ADCC and CDC reduction **G1v21** CH2 Y15.1, T16, E18 Half-life extension	
sarilumab (mAbID 400)		IgG1-kappa		RA|Ankylosing spondylitis (AS)|Uveitis Polymyalgia Rheumatica (PMR)		FDA: May 2017
vobarilizumab (mAbID 523)		VH–VH’		RA|SLE| Inflammatory conditions		
clazakizumab (mAbID 414)	IL6	IgG1-kappa	Blocking ** Immunosuppressant	CD|RA|Psoriatic arthritis (PSA)|AMR|Cancers, lung	**G1v29** CH2 A84.4 No-glycosylation site ADCC reduction	FDA: August 2019
olokizumab *** (mAbID 353)		IgG4-kappa		Autoimmune diseases|CD|RA	**G4v5** h P10 Half-IG exchange reduction	
siltuximab (mAbID 297)		IgG1-kappa		MM|Multicentric Castleman’s disease (MCD)|Renal cell carcinoma (RCC)|Neoplasms		EMA: May 2014 FDA: April 2014
sirukumab *** (mAbID 384)		IgG1-kappa		RA (Despite Methotrexate Therapy)| Lupus nephritis|Juvenile Idiopathic Arthitis (JIA), pediatric|Giant cell arteritis|PMR		FDA: July 2017
ziltivekimab *** (mAbID 979)		IgG1-kappa		Anemia	**G1v21** CH2 Y15.1, T16, E18 Half-life extension	
mezagitamab * (mAbID 882)	CD38	IgG1-lambda	Blocking ** Immunosuppressant, Fc-effector function	MM|SLE|MG		FDA: January 2019
daratumumab * (mAbID 301)		IgG1-kappa		MM|AL amyloidosis|Myeloma, multiple (MM), recurrent or refractory (in combination with lenalidomide and dexamethasone or bortezomib and dexamethasone)		FDA: November 2015
felzartamab (mAbID 1011)		IgG1-lambda2		MM		
isatuximab (mAbID 539)		IgG1-kappa		MM|Hematologic malignancies		FDA: May 2014

* MAbs studied for MG. ** The mechanism of action is described in bold, and its effects in italics. *** Monoclonal antibodies with an MOA suggested by IMGT owing to a lack of scholarly studies giving proof of their preclinical effects. Their MOA may evolve as new data emerge. IMGT suggestion is based on (i) the function of the mAb target in the cancerous environment and (ii) the analysis of their Fc region, when possible.

### 3.5. Monoclonal Antibodies to Inactivate T-Cell Functions

Drugs that block T-cell activity or cytokines may be another effective therapy option for MG, as in this disease, T cells induce B cell growth and differentiation into plasma cells. Plasma cell differentiation and development are induced by proinflammatory cytokines and chemokines, which are essential components in the pathophysiology of MG. CD40 cell surface receptor is expressed on all mature B cells but not on plasma cells and is also expressed on dendritic cells, monocytes, endothelial and epithelial cells [70]. In addition, the CD40-ligand (CD40LG/CD154), a member of the TNF family, is also expressed more widely than activated CD4+ T cells only [71]. The association between CD40 and its ligand CD40LG in T cells encourages the release of cytokines, the dendritic cells’ activation, and an overall increase in the immune response [72]. Disrupting this signaling pathway may potentially reduce the production of proinflammatory cytokines T helper (TH) function and inhibit macrophage activation.

IMGT/mAb-DB has fourteen mAbs assigned by INN against CD40, mostly for the oncology clinical domain, with only three involved in immunology and one related to MG (Table 4). Iscalimab (mAbID 799), a nondepleting anti-CD40 monoclonal antibody, binds to CD40 molecule and blocks CD40-CD40LG co-stimulatory pathway signaling, resulting in hindering humoral and cellular immune responses. Iscalimab is an IgG1-kappa aglycosylated antibody via one mutation in the CH2 domain (G1v29 CH2 A84.4), resulting in loss of FcγR binding ability to prevent immune cell depletion. Phase II trials showed good results concerning the safety of iscalimab; however, there was no improvement regarding patients with generalized AChR and MuSK antibody-positive MG (clinicaltrials.gov NCT02565576).

Interleukin 6 (IL6) is a pleiotropic cytokine that plays an important role in the immune response by regulating cell proliferation and differentiation [73]. The IL6 receptor (IL6R) is a protein complex that consists of IL6 and a receptor subunit, interleukin 6 signal transducer (IL6ST/gp130), that is also shared by many other cytokines. IL6R binds to IL6 with low affinity, and interaction with IL6ST leads to the regulation of the immune response, hematopoiesis, and acute-phase reactions [74,75]. The receptor has two isoforms; the membrane-bound IL6R induces an anti-inflammatory “classic signaling” that is mostly regenerative, and the soluble form of IL6R that acts as an agonist of IL6 activity, binds to IL6ST on cell surfaces and induces a process called “trans-signaling.” The proinflammatory characteristics of this interleukin are mediated by the soluble form of IL6R, which also plays a significant role in the development of chronic inflammatory disorders [76]. Dysregulated production of IL6 and IL6R has been suggested to contribute to the pathogenesis of many diseases, such as prostate cancer, multiple myeloma, and autoimmune diseases [77]. 

**Figure 7:**
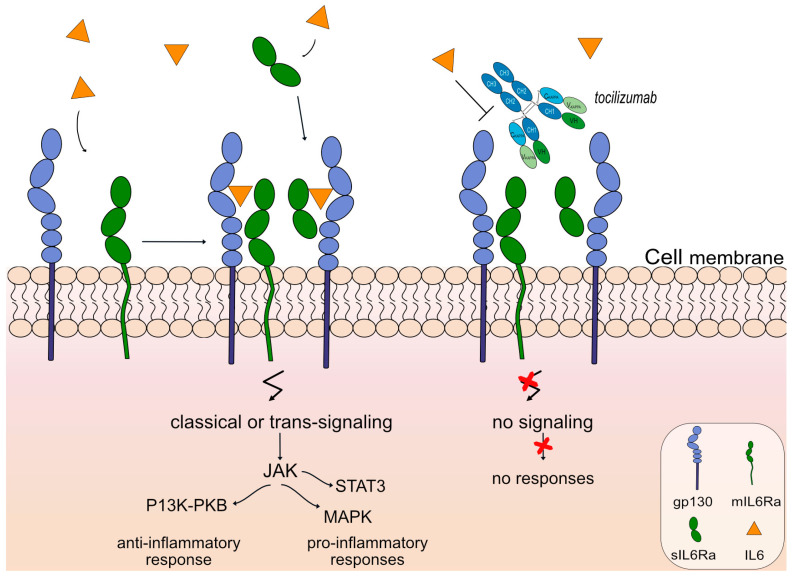
Tocilizumab mechanism of action. Tocilizumab targets and binds to IL6R. Once bound to its target, tocilizumab inhibits IL6 from binding to IL6R; therefore, classic and trans-signaling activation is limited through the Janus kinase (JAK) inhibitors and prohibits anti-inflammatory (phosphoinositide-3-kinase (PI3K) and protein kinase B/PKB pathway) and proinflammatory responses (Signal Transducer And Activator Of Transcription 3-STAT3 and MAPK (mitogen-activated protein kinase) pathway) respectively. Tocilizumab is an IgG1-kappa antibody that triggers antibody-dependent cellular cytotoxicity (ADCC). Mechanism of action: Blocking. Effect: Immunosuppressant, Fc-effector function. (mAbID 96).

An anti-IL6 receptor antibody, tocilizumab (ACTEMRA^®^, RoACTEMRA^®^, mAbID 96), has been approved to treat several autoimmune diseases, such as rheumatoid arthritis [78], systemic juvenile idiopathic arthritis [79] and systemic sclerosis [80] by the FDA and EMA. Tocilizumab targets and binds to IL-6R and blocks its binding to IL6, limiting signal activation and, therefore, there is no regulation of the immune response, no acute-phase reactions, and no hematopoiesis. The restricted expression of IL6R limits classic IL6 signaling and trans-signaling, prohibiting anti-inflammatory and proinflammatory responses, respectively, as shown in Figure 7. Tocilizumab is an IgG1-kappa humanized mAb with the ability to bind to FcγRIIIa and induce ADCC. Its mechanism of action is blocking with an immunosuppressant effect and Fc-effector function. Currently, there is an ongoing phase 3 clinical trial (clinicaltrials.gov NCT05716035) as an extension study for the participants who previously completed study tMG (clinicaltrials.gov NCT05067348) to evaluate the safety and efficacy of the mAb for gMG patients.

Likewise, satralizumab (ENSPRYNG™, mAbID 586), an IgG2 antibody against IL6R with prolonged half-life, has been recently approved as a therapeutic option for neuromyelitis optica [81] and there is currently a phase III clinical trial for MG recruiting to evaluate the efficacy, safety and pharmacokinetics/pharmacodynamics in patients with generalized MG (clinicaltrials.gov NCT04963270). Satralizumab presents the same mechanism as tocilizumab with limited FcγR binding due to its IgG2 subclass.

## 4. Discussion and Conclusions

New biological agents have brought considerable changes in the treatment of Myasthenia Gravis. Monoclonal antibodies have been shown to be used as treatments for a variety of diseases. A series of mAbs have been used for treating MG patients, such as rituximab, eculizumab, efgartigimod alfa, and rozanolixizumab, which is the only FDA-approved treatment in adults for both anti-AChR and anti-MuSK antibody-positive gMG. 

Multiple new options have been discussed through numerous studies, as analyzed in this study, and there have been positive results for a few of these options in clinical trials, while there are still several studies and trials ongoing. New therapeutic targets include FcγR, C5, and molecules such as CD38, CD40, CD19, and interleukin-6 and IL-6 receptors. Efgartigimod alfa and rozanolixizumab act on the FcRn target, leading to increased IgG catabolism and have; as a result, a reduction in the available IgGs with a neutralizing MOA and FcRn inhibitor effect. Eculizumab binds uniquely to C5 and prevents the enzymatic hydrolysis of the molecule to C5a and C5b by blocking MOA with a complement inhibitor effect, while rituximab targets MS4A1 and induces ADCC, CDC, and apoptosis of CD20+ cells, resulting in depletion of B-cells and has a blocking MOA with immunosuppressant and Fc-effector effect.

The changing paradigm in MG has a global impact, and drugs that are currently administered or studied for other autoimmune diseases could prove to be beneficial in the case of MG as well. Emphasis is given to therapies that target B-cells, either directly or indirectly, as well as the complement, which plays a significant role in the disease. Much of the evidence for the importance of the complement in MG comes from animal models of experimental MG that have shown that genetically deficient complement mice present resistibility or less susceptibility to experimental autoimmune MG (EAMG). The use of anti-complement antibodies has shown that it protects mice from developing typical symptoms, such as muscle weakness [31,82]. 

The introduction of mAbs has altered the field of therapeutic options not only in MG but in many other autoimmune diseases. Conventional treatments are, in the largest percentage of patients, effective, but in the case of refractory MG, new options with biological agents are needed [20]. Novel therapeutics present many advantages but have some limitations, such as limited safety data at the moment. It is important to consider the mechanism of action of each new mAb in association with the immunopathogenesis of each of the subtypes of MG (anti-AChR, anti-MuSK, anti-agrin, seronegative, etc.) in order to integrate the new monoclonal antibodies as a viable and safe option in the therapy decision process. The high cost of some of these new treatment options is another point that needs evaluation, as cost-effectiveness needs to be taken into consideration when developing novel biological agents [83].

Each mAb has a different target and different therapeutic effects. It is crucial to meticulously choose the most suitable mAb for each patient to ensure the best prognosis. In the future, mAbs that have been or are currently being investigated for other autoimmune diseases with auto-antibodies production could also be explored as potential treatments for MG (Table 4). MG treatment with various monoclonal antibodies has not yet been the subject of a comparative study, but a network meta-analysis compares the efficiency and safety of different mAbs through randomized controlled trials [84]. Clinical studies are needed to compare the adverse effects and the efficacy of the different possible mAb treatments, as they are promising emerging therapies. In the treatment of cancer, combination immunotherapies have been shown to have greater efficacy and durability [85].

Personalized treatment is a promising path for the future in order to provide patients with chronic autoimmune diseases a better quality of life with minimal risks when it comes to treatment options. Enhancing our understanding of the disease itself and the intricate mechanisms underlying the effectiveness of new antigen-directed treatments is significant for the development of personalized treatment approaches. By delving deeper into the pathogenicity of MG, comprehensive guidelines could be created that address the full spectrum of the disease. These guidelines should provide healthcare professionals with detailed recommendations for diagnosis, treatment, and disease management tailored to the specific needs of each individual patient by ensuring that treatments address the underlying mechanisms involved. Advancing our knowledge of MG and the mechanisms of action of monoclonal antibodies is essential for catering to the unique needs of MG patients. By integrating a standardized description of the modes of action and effects of monoclonal antibodies in MG into IMGT/mAb-DB, IMGT^®^ offers a more complete understanding of how these mAbs function. IMGT^®^ continues standardized efforts to characterize the mechanisms of action of mAbs targeting various targets in autoimmune diseases, as it was performed with targets in cancer treatment [24], and the goal is to cover the complete IMGT/mAb-DB.

## Figures and Tables

**Table 1:** Blocking mAbs targeting C5 molecule available in IMGT/mAb-DB investigated for the treatment of MG.

INN mAbs	Receptor Identification	IMGT Variants	IMGT MOA *	Clinical Trials for MG
eculizumab	IgG2–G4-kappa	-	Blocking * *Complement Inhibitor*	Phase III (NCT03759366|NCT02301624)|NCT01997229)| Phase II (NCT00727194 Observational (NCT04202341)
ravulizumab	IgG2–G4-kappa	G4v24 CH3 L107, S114 Half-life extension		Phase III (NCT05644561|NCT03920293) Observational (NCT04202341)
gefurulimab	VH-VH’	-		Phase III (NCT05556096)
pozelimab	IgG4-kappa	G4v5 h P10 Half-IG exchange reduction		Phase III (NCT05070858)

* The mechanism of action is described in bold, and its effects in italics.

**Table 2:** Neutralizing mAbs targeting FCGRT available in IMGT/mAb-DB investigated for MG treatment.

INN mAbs	Receptor Identification	IMGT Variants	IMGT MOA *	Clinical Trials for MG
efgartigimod alfa	IgG1-Fc fragment	G1v46 CH3 K113, F114 increase FcRn binding	Neutralizing * *FcRn inhibitor*	Phase III (NCT04980495)
batoclimab	IgG1	G1v14 CH2 A1.3, A1.2 ADCC and CDC reduction		Phase III (NCT05403541)
rozanolixizumab	IgG4-kappa	G4v5 h P10 Half-IG exchange reduction		Phase III (NCT03971422|NCT05681715|NCT04124965)
nipocalimab	IgG1-lambda3	G1v29 A84.4 No N-glycosylation site ADCC reduction		Phase III (NCT05265273|NCT04951622)
orilanolimab	IgG4-kappa	G4v5 h P10 Half-IG exchange reduction		Phase I (discontinued)

* The mechanism of action is described in bold, and its effects in italics.

**Table 3:** Blocking mAbs targeting MSA4A1 and CD19 available in IMGT/mAb-DB that have been investigated for the treatment of MG.

Target	INN mAbs	Receptor Identification	IMGT MOA *	Clinical Trials for MG
MS4A1	rituximab	IgG1-kappa	Blocking * *Immunosuppressant,* *Fc-effector function*	Phase III NCT05868837|NCT05332587
	ofatumumab	IgG1-kappa		-
CD19	inebilizumab	IgG1-kappa		Phase III NCT04524273

* The mechanism of action is described in bold, and its effects in italics.

## Data Availability

The datasets analyzed in the current study are available in IMGT/mAb-DB, The International ImMunoGeneTics Information System^®^.

## Abbreviations

AChEI	acetylcholinesterase inhibitor
AChR	acetylcholine receptor
ADCC	antibody-dependent cytotoxicity
ADCP	antibody-dependent cellular phagocytosis
aHUS	atypical hemolytic uremic syndrome
C5	complement 5
CD19	cluster of differentiation 19
CD38	cluster of differentiation 38
CD40	cluster of differentiation 40
CDC	complement-dependent cytotoxicity
EAMG	experimental
EMA	European Medicines Agency
EOMG	early onset Myasthenia Gravis
FcRn	fragment crystallizable neonatal receptor
FcγR	Fc gamma (γ) receptors
FDA	U.S. Food and Drug Administration
gMG	generalized Myasthenia Gravis
HGNC	HUGO Gene Nomenclature Committee
Ig	immunoglobulin
IL6	interleukin 6
IL6ST	interleukin 6 signal transducer
IMGT^®^	the international ImMunoGeneTics information system^®^
INN	international nonproprietary name
LOMG	late-onset myasthenia gravis
LRP4	lipoprotein related protein 4
mAbs	monoclonal antibodies
MAC	membrane attack complex
MG	Myasthenia Gravis
MOA	mechanism of action
MS	multiple sclerosis
MS4A1	Membrane Spanning 4-Domains A1
MuSK	muscle-specific kinase
NMJ	neuromuscular junction
NMO	neuromyelitis optica
NMOSD	neuromyelitis optica spectrum disorder
PNH	paroxysmal nocturnal hemoglobinuria
RA	rheumatoid arthritis
TCC	terminal complement complex
WHO	world health organization

## References

[1] Verschuuren J.J., Huijbers M.G., Plomp J.J., Niks E.H., Molenaar P.C., Martinez-Martinez P., Gomez A.M., De Baets M.H., Losen M. (2013). Pathophysiology of myasthenia gravis with antibodies to the acetylcholine receptor, muscle-specific kinase and low-density lipoprotein receptor-related protein 4. Autoimmun. Rev..

[2] Paz M.L., Barrantes F.J. (2019). Autoimmune Attack of the Neuromuscular Junction in Myasthenia Gravis: Nicotinic Acetylcholine Receptors and Other Targets. ACS Chem. Neurosci..

[3] Garcia Estevez D.A., Pardo Fernandez J. (2023). Myasthenia gravis. Update on diagnosis and therapy. Med. Clin..

[4] Pechlivanidou M., Ninou E., Karagiorgou K., Tsantila A., Mantegazza R., Francesca A., Furlan R., Dudeck L., Steiner J., Tzartos J. (2023). Autoimmunity to neuronal nicotinic acetylcholine receptors. Pharmacol. Res..

[5] Gasperi C., Melms A., Schoser B., Zhang Y., Meltoranta J., Risson V., Schaeffer L., Schalke B., Kröger S. (2014). Anti-agrin autoantibodies in myasthenia gravis. Neurology.

[6] Gomez A.M., Broeck J.V.D., Vrolix K., Janssen S.P., Lemmens M.A.M., Van Der Esch E., Duimel H., Frederik P., Molenaar P.C., Martínez-Martínez P. (2010). Antibody effector mechanisms in myasthenia gravis-pathogenesis at the neuromuscular junction. Autoimmunity.

[7] Berrih-Aknin S., Le Panse R. (2014). Myasthenia gravis: A comprehensive review of immune dysregulation and etiological mechanisms. J. Autoimmun..

[8] Golfinopoulou R., Papageorgiou L., Efthimiadou A., Bacopoulou F., Chrousos G.P., Eliopoulos E., Vlachakis D. (2021). Clinical Genomic, phenotype and epigenetic insights into the pathology, autoimmunity and weight management of patients with Myasthenia Gravis (Review). Mol. Med. Rep..

[9] Tsiamalos P., Kordas G., Kokla A., Poulas K., Tzartos S.J. (2009). Epidemiological and immunological profile of muscle-specific kinase myasthenia gravis in Greece. Eur. J. Neurol..

[10] Li Y., Peng Y., Yang H. (2023). Serological diagnosis of myasthenia gravis and its clinical significance. Ann. Transl. Med..

[11] Zisimopoulou P., Evangelakou P., Tzartos J., Lazaridis K., Zouvelou V., Mantegazza R., Antozzi C., Andreetta F., Evoli A., Deymeer F. (2014). A comprehensive analysis of the epidemiology and clinical characteristics of anti-LRP4 in myasthenia gravis. J. Autoimmun..

[12] Tzartos J.S., Zhang B., Belimezi M., Ragheb S., Bealmear B., Lewis R.A., Xiong W.-C., Lisak R.P., Tzartos S.J., Mei L. (2012). Autoantibodies to lipoprotein-related protein 4 in patients with double-seronegative myasthenia gravis. Arch. Neurol..

[13] Phillips L.H. (2004). The epidemiology of myasthenia gravis. Semin. Neurol..

[14] Hughes B.W., Moro De Casillas M.L., Kaminski H.J. (2004). Pathophysiology of myasthenia gravis. Semin. Neurol..

[15] Sanders D.B., Wolfe G.I., Narayanaswami P. (2016). International consensus guidance for management of myasthenia gravis: Executive summary. Neurology.

[16] Seybold M.E., Drachman D.B. (1974). Gradually increasing doses of prednisone in myasthenia gravis. Reducing the hazards of treatment. N. Engl. J. Med..

[17] Morren J., Li Y. (2020). Maintenance immunosuppression in myasthenia gravis, an update. J. Neurol. Sci..

[18] Alhaidar M.K., Abumurad S., Soliven B., Rezania K. (2022). Current Treatment of Myasthenia Gravis. J. Clin. Med..

[19] Howard J.F., Barohn R.J., Cutter G.R., Freimer M., Juel V.C., Mozaffar T., Mellion M.L., Benatar M.G., Farrugia M.E., Wang J.J. (2013). A randomized, double-blind, placebo-controlled phase II study of eculizumab in patients with refractory generalized myasthenia gravis. Muscle Nerve.

[20] Legendre C.M., Licht C., Muus P., Greenbaum L.A., Babu S., Bedrosian C., Bingham C., Cohen D.J., Delmas Y., Douglas K. (2013). Terminal complement inhibitor eculizumab in atypical hemolytic-uremic syndrome. N. Engl. J. Med..

[21] Dmytrijuk A., Robie-Suh K., Cohen M.H., Rieves D., Weiss K., Pazdur R. (2008). FDA report: Eculizumab (Soliris) for the treatment of patients with paroxysmal nocturnal hemoglobinuria. Oncologist.

[22] Pittock S.J., Berthele A., Fujihara K., Kim H.J., Levy M., Palace J., Nakashima I., Terzi M., Totolyan N., Viswanathan S. (2019). Eculizumab in Aquaporin-4-Positive Neuromyelitis Optica Spectrum Disorder. N. Engl. J. Med..

[23] Manso T., Folch G., Giudicelli V., Jabado-Michaloud J., Kushwaha A., Ngoune V.N., Georga M., Papadaki A., Debbagh C., Pégorier P. (2021). IMGT^®^ databases, related tools and web resources through three main axes of research and development. Nucleic Acids Res..

[24] Manso T., Kushwaha A., Abdollahi N., Duroux P., Giudicelli V., Kossida S. (2023). Mechanisms of action of monoclonal antibodies in oncology integrated in IMGT/mAb-DB. Front. Immunol..

[25] Ehrenmann F., Kaas Q., Lefranc M.P. (2010). IMGT/3Dstructure-DB and IMGT/DomainGapAlign: A database and a tool for immunoglobulins or antibodies, T cell receptors, MHC, IgSF and MhcSF. Nucleic Acids Res..

[26] Lefranc M.P., Lefranc G. (2022). IMGT ((R)) Nomenclature of Engineered IGHG Variants Involved in Antibody Effector Properties and Formats. Antibodies.

[27] Engel A.G., Lambert E.H., Howard F.M. (1977). Immune complexes (IgG and C3) at the motor end-plate in myasthenia gravis: Ultrastructural and light microscopic localization and electrophysiologic correlations. Mayo Clin. Proc..

[28] Romi F., Kristoffersen E.K., Aarli J.A., Gilhus N.E. (2005). The role of complement in myasthenia gravis: Serological evidence of complement consumption in vivo. J. Neuroimmunol..

[29] Kaminski H.J., Kusner L.L., Richmonds C., Medof M.E., Lin F. (2006). Deficiency of decay accelerating factor and CD59 leads to crisis in experimental myasthenia. Exp. Neurol..

[30] Howard J.F. (2018). Myasthenia gravis: The role of complement at the neuromuscular junction. Ann. N. Y. Acad. Sci..

[31] Mantegazza R., Vanoli F., Frangiamore R., Cavalcante P. (2020). Complement Inhibition for the Treatment of Myasthenia Gravis. Immunotargets Ther..

[32] Rother R.P., Rollins S.A., Mojcik C.F., Brodsky R.A., Bell L. (2007). Discovery and development of the complement inhibitor eculizumab for the treatment of paroxysmal nocturnal hemoglobinuria. Nat. Biotechnol..

[33] Nicholas S.R. (2022). Sanderson, Complement and myasthenia gravis. Mol. Immunol..

[34] Stern R.M., Connell N.T. (2019). Ravulizumab: A novel C5 inhibitor for the treatment of paroxysmal nocturnal hemoglobinuria. Ther. Adv. Hematol..

[35] Vu T., Ortiz S., Katsuno M., Annane D., Mantegazza R., Beasley K.N., Aguzzi R., Howard J.F. (2023). Ravulizumab pharmacokinetics and pharmacodynamics in patients with generalized myasthenia gravis. J. Neurol..

[36] Kulasekararaj A.G., Hill A., Rottinghaus S.T., Langemeijer S., Wells R., Gonzalez-Fernandez F.A., Gaya A., Lee J.W., Gutierrez E.O., Piatek C.I. (2019). Ravulizumab (ALXN1210) vs eculizumab in C5-inhibitor-experienced adult patients with PNH: The 302 study. Blood.

[37] Jang J.H., Weyne J., Chaudhari U., Harari O., Miller J., Dain B., Meagher K.A., Rodgers M.L., Perlee L., Morton L. (2021). Pozelimab, a Human Monoclonal Antibody Against Complement Factor C5, Provided Inhibition of Intravascular Hemolysis in Patients with Paroxysmal Nocturnal Hemoglobinuria. Blood.

[38] Latuszek A., Liu Y., Olsen O., Foster R., Cao M., Lovric I., Yuan M., Liu N., Chen H., Zhang Q. (2020). Inhibition of complement pathway activation with Pozelimab, a fully human antibody to complement component C5. PLoS ONE.

[39] Pyzik M., Sand K.M., Hubbard J.J., Andersen J.T., Sandlie I., Blumberg R.S. (2019). The Neonatal Fc Receptor (FcRn): A Misnomer?. Front. Immunol..

[40] Dalakas M.C., Spaeth P.J. (2021). The importance of FcRn in neuro-immunotherapies: From IgG catabolism, FCGRT gene polymorphisms, IVIg dosing and efficiency to specific FcRn inhibitors. Ther. Adv. Neurol. Disord..

[41] Gable K.L., Guptill J.T. (2019). Antagonism of the Neonatal Fc Receptor as an Emerging Treatment for Myasthenia Gravis. Front. Immunol..

[42] Bril V., Drużdż A., Grosskreutz J., Habib A.A., Mantegazza R., Sacconi S., Utsugisawa K., Vissing J., Vu T., Boehnlein M. (2023). Safety and efficacy of rozanolixizumab in patients with generalised myasthenia gravis (MycarinG): A randomised, double-blind, placebo-controlled, adaptive phase 3 study. Lancet Neurol..

[43] Iorio R., Damato V., Alboini P.E., Evoli A. (2015). Efficacy and safety of rituximab for myasthenia gravis: A systematic review and meta-analysis. J. Neurol..

[44] Tannemaat M.R., Verschuuren J. (2020). Emerging therapies for autoimmune myasthenia gravis: Towards treatment without corticosteroids. Neuromuscul. Disord..

[45] Saccà F., Pane C., Espinosa P.E., Sormani M.P., Signori A. (2023). Efficacy of innovative therapies in myasthenia gravis: A systematic review, meta-analysis and network meta-analysis. Eur. J. Neurol.

[46] Hehir M.K., Li Y. (2022). Diagnosis and Management of Myasthenia Gravis. Continuum.

[47] Zouvelou V., Psimenou E. (2022). Double Seropositive Myasthenia Gravis Successfully Treated with Rituximab. J. Clin. Neuromuscul. Dis..

[48] Forsthuber T.G., Cimbora D.M., Ratchford J.N., Katz E., Stüve O. (2018). B cell-based therapies in CNS autoimmunity: Differentiating CD19 and CD20 as therapeutic targets. Ther. Adv. Neurol. Disord..

[49] Nair S.S., Jacob S. (2023). Novel Immunotherapies for Myasthenia Gravis. Immunotargets Ther..

[50] Tedder T.F., Klejman G., Schlossman S.F., Saito H. (1989). Structure of the gene encoding the human B lymphocyte differentiation antigen CD20 (B1). J. Immunol..

[51] Tedder T.F., Boyd A.W., Freedman A.S., Nadler L.M., Schlossman S.F. (1985). The B cell surface molecule B1 is functionally linked with B cell activation and differentiation. J. Immunol..

[52] Léveillé C., Al-Daccak R., Mourad W. (1999). CD20 is physically and functionally coupled to MHC class II and CD40 on human B cell lines. Eur. J. Immunol..

[53] Boyles J.S., Sadowski D., Potter S., Vukojicic A., Parker J., Chang W.Y., Ma Y.L., Chambers M.G., Nelson J., Barmettler B. (2023). A nondepleting anti-CD19 antibody impairs B cell function and inhibits autoimmune diseases. JCI Insight.

[54] Tandan R., Hehir M.K., Waheed W., Howard D.B. (2017). Rituximab treatment of myasthenia gravis: A systematic review. Muscle Nerve.

[55] Narayanaswami P., Sanders D.B., Wolfe G., Benatar M., Cea G., Evoli A., Gilhus N.E., Illa I., Kuntz N.L., Massey J. (2021). International Consensus Guidance for Management of Myasthenia Gravis: 2020 Update. Neurology.

[56] Łosińska K., Korkosz M., Pripp A.H., Haugeberg G. (2023). Real-world experience of rituximab biosimilar GP2013 in rheumatoid arthritis patients naive to or switched from reference rituximab. Rheumatol. Int..

[57] Chmielewska N., Szyndler J. (2023). Targeting CD20 in multiple sclerosis—Review of current treatment strategies. Neurol. Neurochir. Pol..

[58] Rual C., Biotti D., Lepine Z., Delourme A., Le Berre J., Treiner E., Ciron J. (2023). 2 grams versus 1 gram rituximab as maintenance schedule in multiple sclerosis, neuromyelitis optica spectrum disorders and related diseases: What B-cell repopulation data tell us. Mult. Scler. Relat. Disord..

[59] Waters M.J., Field D., Ravindran J. (2019). Refractory myasthenia gravis successfully treated with ofatumumab. Muscle Nerve.

[60] Sánchez-Tejerina D., Sotoca J., Llaurado A., López-Diego V., Juntas-Morales R., Salvado M. (2022). New Targeted Agents in Myasthenia Gravis and Future Therapeutic Strategies. J. Clin. Med..

[61] Menon D., Bril V. (2022). Pharmacotherapy of Generalized Myasthenia Gravis with Special Emphasis on Newer Biologicals. Drugs.

[62] Frampton J.E. (2020). Inebilizumab: First Approval. Drugs.

[63] Viela Bio (2021). A Randomized, Double-Blind, Multicenter, Placebocontrolled Phase 3 Study with Open-Label Period to Evaluate the Efficacy and Safety of Inebilizumab in Adults with Myasthenia Gravis.

[64] Gallagher S., Turman S., Yusuf I., Akhgar A., Wu Y., Roskos L.K., Herbst R., Wang Y. (2016). Pharmacological profile of MEDI-551, a novel anti-CD19 antibody, in human CD19 transgenic mice. Int. Immunopharmacol..

[65] Chen D., Gallagher S., Monson N.L., Herbst R., Wang Y. (2016). Inebilizumab, a B Cell-Depleting Anti-CD19 Antibody for the Treatment of Autoimmune Neurological Diseases: Insights from Preclinical Studies. J. Clin. Med..

[66] Tenca C., Merlo A., Zarcone D., Saverino D., Bruno S., De Santanna A., Ramarli D., Fabbi M., Pesce C., Deaglio S. (2003). Death of T cell precursors in the human thymus: A role for CD38. Int. Immunol..

[67] Scheibe F., Ostendorf L., Prüss H., Radbruch H., Aschman T., Hoffmann S., Blau I., Meisel C., Alexander T., Meisel A. (2022). Daratumumab for treatment-refractory antibody-mediated diseases in neurology. Eur. J. Neurol..

[68] Field-Smith A., Morgan G.J., Davies F.E. (2006). Bortezomib (Velcadetrade mark) in the Treatment of Multiple Myeloma. Ther. Clin. Risk Manag..

[69] Gomez A.M. (2011). Proteasome inhibition with bortezomib depletes plasma cells and autoantibodies in experimental autoimmune myasthenia gravis. J. Immunol..

[70] Clark E.A. (1990). CD40: A cytokine receptor in search of a ligand. Tissue Antigens.

[71] van Kooten C., Banchereau J. (2000). CD40-CD40 ligand. J. Leukoc. Biol..

[72] Huda R., Tuzun E., Christadoss P. (2014). Targeting complement system to treat myasthenia gravis. Rev. Neurosci..

[73] Hunter C.A., Jones S.A. (2015). IL-6 as a keystone cytokine in health and disease. Nat. Immunol..

[74] Kang S., Tanaka T., Narazaki M., Kishimoto T. (2019). Targeting Interleukin-6 Signaling in Clinic. Immunity.

[75] Spencer S., Köstel Bal S., Egner W., Lango Allen H., Raza S.I., Ma C.A., Gürel M., Zhang Y., Sun G., Sabroe R.A. (2019). Loss of the interleukin-6 receptor causes immunodeficiency, atopy, and abnormal inflammatory responses. J. Exp. Med..

[76] Garbers C., Thaiss W., Jones G.W., Waetzig G.H., Lorenzen I., Guilhot F., Lissilaa R., Ferlin W.G., Grötzinger J., Jones S.A. (2011). Inhibition of classic signaling is a novel function of soluble glycoprotein 130 (sgp130), which is controlled by the ratio of interleukin 6 and soluble interleukin 6 receptor. J. Biol. Chem..

[77] Hirano T. (2021). IL-6 in inflammation, autoimmunity and cancer. Int. Immunol..

[78] Biggioggero M., Crotti C., Becciolini A., Favalli E.G. (2019). Tocilizumab in the treatment of rheumatoid arthritis: An evidence-based review and patient selection. Drug Des. Dev. Ther..

[79] Yan X., Tang W., Zhang Z., Zhang Y., Luo C., Tang X. (2021). Tocilizumab in Systemic Juvenile Idiopathic Arthritis: Response Differs by Disease Duration at Medication Initiation and by Phenotype of Disease. Front. Pediatr..

[80] Khanna D., Lin C.J.F., Furst D.E., Goldin J., Kim G., Kuwana M., Allanore Y., Matucci-Cerinic M., Distler O., Shima Y. (2020). Tocilizumab in systemic sclerosis: A randomised, double-blind, placebo-controlled, phase 3 trial. Lancet Respir. Med..

[81] Traboulsee A., Greenberg B.M., Bennett J.L., Szczechowski L., Fox E., Shkrobot S., Yamamura T., Terada Y., Kawata Y., Wright P. (2020). Safety and efficacy of satralizumab monotherapy in neuromyelitis optica spectrum disorder: A randomised, double-blind, multicentre, placebo-controlled phase 3 trial. Lancet Neurol..

[82] Kusner L.L., Kaminski H.J., Soltys J. (2008). Effect of complement and its regulation on myasthenia gravis pathogenesis. Expert Rev. Clin. Immunol..

[83] Alabbad S., AlGaeed M., Sikorski P., Kaminski H.J. (2020). Monoclonal Antibody-Based Therapies for Myasthenia Gravis. BioDrugs.

[84] Song Z., Zhang J., Meng J., Jiang G., Yan Z., Yang Y., Chen Z., You W., Wang Z., Chen G. (2021). Different Monoclonal Antibodies in Myasthenia Gravis: A Bayesian Network Meta-Analysis. Front. Pharmacol..

[85] Marshall H.T., Djamgoz M.B.A. (2018). Immuno-Oncology: Emerging Targets and Combination Therapies. Front. Oncol..

